# Atmospheric pressure plasma liquid assisted deposition of polydopamine/acrylate copolymer on zirconia (Y-TZP) ceramics: a biocompatible and adherent nanofilm†

**DOI:** 10.1039/d1ra02054d

**Published:** 2021-05-11

**Authors:** Ľudmila Hodásová, Robert Quintana, Urszula Czuba, Luis J. del Valle, Gemma Fargas, Carlos Alemán, Elaine Armelin

**Affiliations:** Departament d'Enginyeria Química, Universitat Politècnica de Catalunya, Campus Diagonal Besòs (EEBE) C/ Eduard Maristany, 10-14, Building I, 2nd Floor Barcelona 08019 Spain elaine.armelin@upc.edu; Departament de Ciència i Enginyeria de Materials, Universitat Politècnica de Catalunya, Campus Diagonal Besòs – EEBE C/ Eduard Maristany, 10-14, Building I, 1st Floor Barcelona 08019 Spain; Barcelona Research Center for Multiscale Science and Engineering, Universitat Politècnica de Catalunya, Campus Diagonal Besòs (EEBE) C/ Eduard Maristany, 10-14, Building I, Basement Floor Barcelona 08019 Spain; Luxembourg Institute of Science and Technology (LIST), Materials Research and Technology Department L-4422 Belvaux Luxembourg roberto.quintana@list.lu

## Abstract

Polydopamine–ethylene glycol dimethacrylate copolymer is a biocompatible coating with cell adhesion promotion and antibiofilm properties. This copolymer has been successfully applied on metallic implants, such as stainless steel and titanium implants, using several deposition techniques (*e.g*. layer-by-layer, silane activation, chemical vapor deposition, or liquid-assisted plasma polymerization). However, its application in zirconia ceramic materials, which are widely used in dentistry and medicine, has never been described. In this work, polydopamine–ethylene glycol dimethacrylate copolymer has been deposited on ultra-smooth surfaces of yttria-stabilized zirconia discs (average roughness = 2.08 ± 0.08 nm) by using liquid-assisted atmospheric-pressure plasma-induced polymerization (LA-APPiP). After the polymerization, the nanometric coating (250 nm, measured by ellipsometry) had an average roughness of 79.85 ± 13.71 nm and water contact angle of 57.8 ± 2.2 degrees, consistent with the highly hydrophilic nature of the biocompatible copolymer, if compared to the pristine zirconia (72.7 ± 2.0 degrees). The successful covalent bonding of the copolymer with the zirconia surface, thanks to the previous activation of the substrate with oxygen plasma, was proved by X-ray photoelectron spectroscopy (XPS). The polymer composition has been investigated by XPS and Raman spectroscopies. The LA-APPiP technique has been proved to be an excellent method to produce homogenous films without the need to employ solvents and further purification steps. The new copolymer film allows the uniform growth of human osteoblast-like MG-63 cells, after 7 days of cell culture, as observed by fluorescence microscopy.

## Abstract 2

Polydopamine–ethylene glycol dimethacrylate copolymer is a biocompatible coating with cell adhesion promotion and antibiofilm properties.

## Graphical abstract

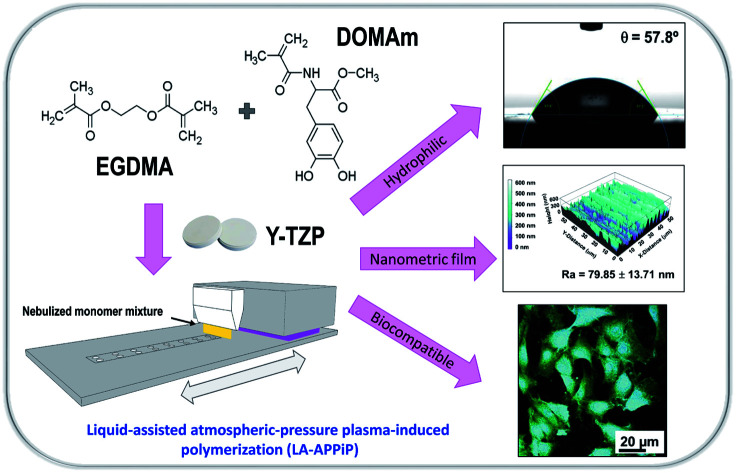

## 1. Introduction

The use of bioceramic materials in dentistry and medicine has been increasing in the past decades due to the facile modulation of their dynamic properties and the simplicity of the required fabrication processes.^1^ For instance, 3D-printing technology is gaining momentum for ceramic tool production and has partially replaced the conventional cold isostatic pressing and sintering process.^2–6^ Among ceramic compounds, yttria-stabilized tetragonal zirconia polycrystal (Y-TZP) has shown a great promise in many applications, such as hip joint replacement, dental implants, and long-span bridges.^7–9^ Moreover, Y-TZP biomedical implants offer important advantages, which can be summarized as follows: (i) high affinity to bone tissue; (ii) biocompatible and non-carcinogenic properties; (iii) proved nucleation site service for calcium-based mineral growth, essential for bone restoration; (iv) avoidance of bluish discoloration, usually observed in titanium prosthesis; and (v) light-weight when compared to metal implants and not prone to corrode if compared to other metals as well.

Particularly, in the dental industry, there is a significant interest in producing surface treatments on zirconia-based substrates to achieve enhanced fibroblast adherence, decrease biofilm formation, and focus on therapeutic aims.^10–12^ The objective is to improve osseointegration and antimicrobial activity in order to reduce the percentage of biomaterial rejections once implanted.^13–16^

In addition to traditional surface pre-treatments, like chemical etching, grit blasting or machining, laser ablation, UV-light radiation,^11,15,17–19^ and coating deposition by using dip-coating methods, as the sol–gel technology,^20–22^ there is nowadays a plethora of friendly and advantageous bottom-up approaches that lead to the obtaining of hybrid materials of great interest. In this sense, atmospheric plasma technology has proved to be a time- and resource-efficient one-step process able to control the hydrophobicity of solid surfaces and useful to completely eradicate the use of solvents and co-additives in the preparation of organic and inorganic cladding hybrid systems.^18,23–25^

In this work, for the first time, organically modified Y-TZP discs containing polydopamine and acrylate biocompatible polymer were successfully prepared by applying liquid-assisted atmospheric-pressure plasma-induced polymerization (LA-APPiP). The nanocoating was designed to contain polydopamine molecules,^26–28^ which promote surface adhesion due to catecholamine groups^29^ and biominerals formation,^30,31^ such as calcium phosphates and hydroxyapatites, that are responsible for the rapid osseointegration of medical implants.^32,33^ Moreover, the acrylate polymer, ethylene glycol dimethacrylate (EGDMA), exhibits biocompatibility, insignificant cytotoxicity, and is extendedly used in the dental field as an adhesive between the titanium screw and the ceramic crown of a dental implant.^34,35^ For instance, Lee and co-workers^36^ have demonstrated the enhanced biocompatibility of the zirconia surface modified with 3,4-dihydroxy-l-phenylalanine (l-DOPA) films. Those authors employed an aqueous base solution (dip-coating method) for the zirconia surface modification. The chemical structure of the coating and the film topography were evaluated by X-ray photoelectron spectroscopy (XPS) and atomic force microscopy (AFM), respectively. Additionally, hybrid organic–inorganic surface materials are usually fabricated by layer-by-layer (LbL) assembly.^37^ However, such technology is being disused due to both: (i) the high amount of raw material and solvents resources needed; and (ii) the long synthesis process, which is not fully cost-effective and environmentally compliant for market applications.

Based on the abovementioned drawbacks, LA-APPiP represents a powerful alternative for Y-TZP surface functionalization. Moreover, the dopamine and the monomers supplied for the copolymer preparation are not only commercially available but also relatively inexpensive reagents. Therefore, the novelty of the present work relies on the first time successful covalent deposition of polydopamine-*co*-polymethacrylate films on the surface of a ceramic substrate by applying atmospheric plasma deposition. Such technology offers the possibility of fast polymer formation and it is scalable for future commercial uses.

The physical and chemical changes on the Y-TZP surface were approached, before and after oxygen-plasma pre-treatment, and after the nanocoating polymerization by LA-APPiP. The precise characterization of the hybrid material surface chemistry is of paramount importance since its properties determine the possible practical applications in the biomedical field. As the substrate material, zirconia, is widely used in dentistry, for the cell viability and adhesion properties evaluation, MG-63 osteoblast cells were cultured with the samples used in the present study. MG-63 cells are one of the most employed osteoblast-like cells used for *in vitro* biocompatibility approach with biomedical materials, as for example, stainless steel, titanium, zirconia, and methacrylate adhesives used in the dentistry field.^11,20,23,38,39^

## 2. Experimental section

### 2.1. Materials

AMES Group supplied yttria-stabilized zirconia (Y-TZP) rods. The fine-grained zirconia was stabilized with a 2.5% molar of Y_2_O_3_. The material composition and main properties are listed in Table S1.† The zirconia discs (diameter of 8.00 mm and thickness of 2.00 ± 0.01 mm) were cut from rods with IsoMet 4000 linear precision saw from Buehler using a diamond cutting disc, polished until mirror grade with diamond polishing discs using Phoenix 4000 polisher machine from Buehler manufacturer. Then, the samples were cleaned in an ultrasonic bath, first with distilled water (3 times, 5 min per time), then with ethanol absolute (3 times, 5 min per time). The samples were stored in a desiccator until use.

Ethylene glycol dimethacrylate (EGDMA, 98%) was purchased from Sigma-Aldrich and used as received. The monomer methyl-DOPA methacrylamide (DOMAm), methyl 3-(3,4-dihydroxyphenyl)-2-(2-methylprop-2-enamido)propanoate, was kindly provided by Symbiose Biomaterials, Belgium.

Human osteoblast-like MG-63 cells were obtained from American Type Culture Collection (ATCC, USA) for the cell viability study, and Dulbecco's phosphate buffered saline (DPBS) was obtained from Gibco (NY, USA). Milli-Q water was used for the sample's stability coating determination.

### 2.2. Zirconia surface cleaning and activation by atmospheric plasma

**Fig. 1:**
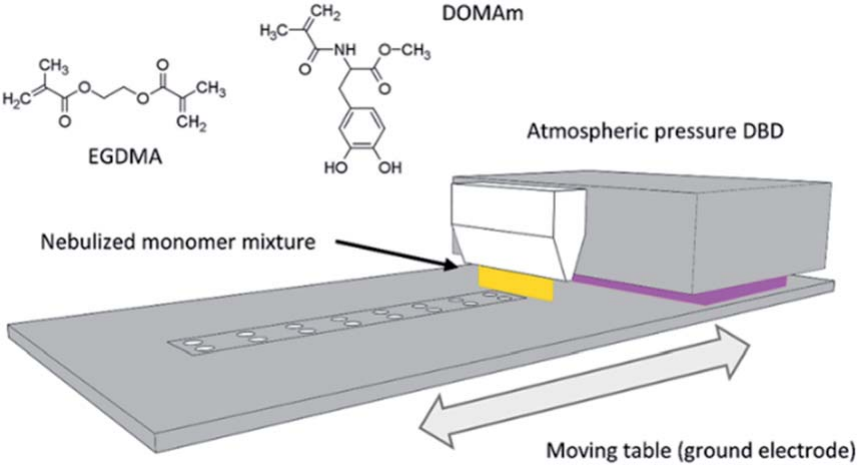
Illustration of dielectric barrier discharge (DBD) deposition setup used to coat the zirconia discs by the Liquid-Assisted Atmospheric Pressure Plasma-induced Polymerization (LA-APPiP) of EGDMA and DOMAm mixtures. A disc holder was inserted in the moving table to keep a constant gap in-between the high voltage electrodes and the table surface.

Before coating, the disc samples were exposed to Ar/O_2_ (5% v/v O_2_) plasma mixture (1.6 W cm^−2^, 20 SLM) for 10 consecutive repeats of 48 s/each repetition, to clean and activate the exposed surface in the same plasma deposition setup described in Section 2.3 and Fig. 1. To assess the effect of the plasma cleaning on the surface of the discs, the standard protocol (SP) was modified by doubling the time (DT) of exposure or by doubling the power (DP) of the plasma treatment.

### 2.3. Plasma deposition of poly(EGDMA-*co*-DOMAm) by LA-APPiP

The atmospheric-pressure plasma-induced polymerization of mixtures of EGDMA and DOMAm was reported elsewhere.^23^ The experimental setup is schematically shown in Fig. 1. The coating deposition proceeded by spraying a very thin liquid layer over the surface of the discs using a stream of very fine droplets of the liquid mixture (0.007 mmol mL^−1^, DOMAm) in nitrogen (4 SLM, 99.999%) produced using a venturi-based nebulization system (VITO) connected to a 3D printed lineal nozzle. The thin liquid layer was then plasma-induced polymerized by two-subsequent brief expositions, only for few seconds, to an atmospheric-pressure direct dielectric discharge (DBD) plasma, generated by 10 kHz sinusoidal electrical excitation (SOFTAL “corona generator 7010R”) providing 1.6 W cm^−2^ and using argon (20 SLM, 99.999%) as plasma gas. The moving table, where the ceramic disc samples (up to 24 units) and a piece of the silicon wafer (as control) were placed, acted as the ground electrode and allowed to repeat the 8 s deposition process until a coating thickness of approximately 250 nm on the silicon control by spectroscopic ellipsometry (VASE-32, J. A. Woollam Co. U.S.) was attained after 150 repeats. The new nanometric film was named as pPoly(EGDMA-*co*-DOMAm), where "p" refers to plasma polymerization.

### 2.4. Zirconia surface and pPoly(EGDMA-*co*-DOMAm) characterization

Several surface characterization techniques were implemented to study the proposed hypothesis. The chemical structure of the plasma polymerized nanocoating (pPoly(EGDMA-*co*-DOMAm)) was evaluated by X-ray photoelectron spectroscopy (XPS) and Raman techniques. The XPS equipment used was a Kratos Axis-Ultra DLD instrument using an Al Kα source (1486.6 eV) with a pass energy of 20 eV and an energy resolution of 0.5 eV. A flooding gun was used to reduce the charging effect on the surface of the sample. Photoelectron emission take-off angle was established at 0° with respect to the surface normal. The C 1s peak with a binding energy of 285 eV was used as the internal reference. The atomic percentage of each element was determined by dividing the peak area of the most intense XPS signal by the corresponding sensitivity factor and by expressing it as a fraction of the sum of all normalized peak areas. High-resolution XPS spectra were acquired by Gaussian/Lorentzian curve fitting after S-shape background subtraction for the following elements: C 1s, O 1s, N 1s, and Zr 3d. Special attention was paid to the analysis of the plasma cleaned sample by transferring it to the XPS equipment just within few minutes after surface treatment.

Raman spectra were acquired in a back-scattering geometry using a Renishaw dispersive Raman microscope spectrometer (model InVia Reflex, GmbH, Germany) and a Renishaw WiRE software. The spectra were acquired with an excitation wavelength of 532 nm line of an Nd:YAG laser. The exposure time was 5 s for each accumulation (×20) at a power of 24 mW. An ×50 long working distance objective was used to focus the laser beam on the sample surface. All Raman spectra were collected in a spectral range from 600 to 4000 cm^−1^ with the same measurement parameters.

Water contact angle (WCA) measurements were taken in a drop shape analyzer (Kruss DSA100). Static WCA values were determined using the sessile water drop method at room temperature and constant relative humidity. Droplet images were recorded at 10 seconds after drop deposition. The contact angle value was extracted from the 2 μL droplet shape and determined using a numerical fit based on the Laplace–Young model in the Advance software (Kruss). Due to the reduced size of the disc surface (approximately 50 mm^2^), only one droplet was deposited on each sample. The values reported for the discs after the ST plasma treatment and coated with the polymer coating are the average of 3 different disc samples. The topography of the surface was analyzed using atomic force microscopy (AFM), employing a Molecular Imaging PicoSPM with NanoScope IV controller under ambient conditions. The tapping mode was operated at constant deflection and the scan speed was 1 Hz for all measurements. Various places on the sample were analyzed using 50 × 50 μm^2^ windows, which correspond to the pictures presented in this work. The Profilmonline software was used to analyze and calculate root-mean-square roughness (*R*_q_) and roughness average (*R*_a_).

Scanning electron microscopy (SEM) was employed for pPoly(EGDMA-*co*-DOMAm) topography analysis. The equipment used is a Focused Ion Beam Zeiss Neon 40 instrument, commercialized by Carl Zeiss (Germany), coupled to a secondary electron beam detector (SE). In order to avoid electron discharge, samples were carbon-coated using Mitek K950 Sputter Coater before analysis. The accelerating voltage for obtaining morphology micrographs was 2 kV.

The coating stability was tested by immersing Y-TZP/pPoly(EGDMA-*co*-DOMAm) discs in a buffer solution (0.01 M PBS, pH 7.4) for 7 days. The experiment was performed at room temperature and the discs were cleaned with Milli-Q water and dried in a vacuum before weighting. The weight of the samples was determined before and after 24 h of solution exposure until 7 days, by using a Sartorius CPA26P Microbalance (5 g × 2 μg/5–21 g × 10 μg). Three replicates were tested. No weight variation was observed. According to the conditions described previously, Raman spectroscopy was used to certify the nanocoating composition after immersion tests.

### 2.5. *In vitro* biocompatibility assays

The MG-63 cells were cultured in Dulbecco's modified Eagle's medium (DMEM with 4500 mg L^−1^ of glucose, 110 mg L^−1^ of sodium pyruvate, and 2 mM of l-glutamine) supplemented with 10% fetal bovine serum (FBS), 50 U cm^−3^ penicillin, 50 mg mL^−1^ streptomycin and l-glutamine 2 mM at 37 °C in a 10% humidified atmosphere of 5% CO_2_ and 95% air. Culture media were changed every two days. For sub-culture, cell monolayers were rinsed with PBS and detached by incubating them with 0.25% trypsin/EDTA for 2–5 min at 37 °C. The incubation was stopped by re-suspending the vials in 5 mL of fresh medium. The cell concentration was determined by counting them with a Neubauer camera and using 4% trypan blue as dye vital.

The zirconia discs with pPoly(EGDMA-*co*-DOMAm) copolymer were placed in tissue culture plates (TCPs) of 24-wells and fixed to the bottom of the plate, with a small drop of silicone (Silbione® Med Adh 4300 RTV, Bluestar Silicones France SAS, Lyon, France). Then, the system was sterilized by exposure to UV light for 15 min. The MG-63 cells were seeded in each TCP well by using the following criteria: (i) for cell adhesion analysis, 100 μL of a suspension containing 2 × 10^4^ cells per well were added to each well, and (ii) for cell proliferation analysis 5 × 10^4^ cells per well were used. After seeding the cells, the plates were incubated for 60 minutes to allow the cell attachment to the material surface. Afterward, 1 mL of culture medium was then added to each well. Quantification of viable cells was performed after 24 h and 7 days to evaluate the cellular adhesion and proliferation, respectively. The control was performed by cell culture on the TCP well, without pPoly(EGDMA-*co*-DOMAm) decorated discs.

The percentage of cells adhered and proliferated was determined through the MTT (3-(4,5-dimethylthiazol-2-yl)-2,5-diphenyltetrazolium bromide) assay.^40^ After 24 h or 7 days, 50 μL of MTT (3 mg mL^−1^) were added to each well in the plates and incubated for 4 h. After that, samples were washed twice with PBS and the specimens were deposited in a new plate. Next, 1 mL of dimethyl sulfoxide (DMSO) was added and the absorbance was measured at 570 nm in a microplate reader (Biochrom EZ-Read 400) after 15 min of gentle stirring. Three replicas were evaluated and the corresponding values were averaged.

To obtain images of the morphology of the cells after the adhesion and proliferation assays, samples were fixed overnight with 2.5% formaldehyde in PBS at 4 °C and then washed five times with PBS. Samples were stained for fluorescence microscopy. Specifically, actin was labeled with green-fluorescent Alexa Fluor Atto-488 phalloidin dye, and the nucleus was labeled with DAPI (4′,6-diamidino-2-phenylindole). Then, the samples were observed in a confocal laser scanning microscope (LSM 900 Zeiss) and photographed with a camera controlled by ZEN 2.6 software (blue edition) (Carl-Zeiss Microscopy GmbH, Jena, Germany).

## 3. Results and discussion

### 3.1. Atmospheric-pressure plasma deposition of poly(EGDMA-*co*-DOMAm) in zirconia substrates activated by oxygen plasma

Previous works demonstrated the applicability of the LA-APPiP deposition method to obtain biocomposites for biomedical applications.^23,24^ In such studies, the surfaces explored were mainly stainless steel, titanium, and silicon wafer-substrates. For example, the method allowed the fast deposition of pine hole-free polymeric coatings functionalized with catechol and quinone groups that were used for the bioactivation of the coating by controlled covalent immobilization of enzymes and biosurfactant proteins. In the present study, for the first time, polydopamine/acrylate copolymer derivative has been used to cover a ceramic substrate used in dentistry applications to improve biocompatibility of ceramic. The process involved a preliminary step of plasma cleaning of the zirconia material to create active sites and modify the surface wettability and, subsequently, chemical polymerization of the biocompatible copolymer and the growth of osteoblastic cells for the evaluation of its *in vitro* biocompatibility response.

The disc topmost surface chemical composition was analyzed by XPS after each step of the deposition process. The survey spectra of the zirconia disc surface before and after O_2_ plasma cleaning and further coated with the polymer layer are shown in Fig. S1.† The presence of the coating on the zirconia disc after plasma deposition is clearly evidenced by the increase in the signals generated from the organic components (C 1s, O 1s, and N 1s, Table S2†). Despite the thickness determined by ellipsometry (250 ± 17 nm), which was much higher than the deep of electron beam penetration (<10 nm) of the XPS equipment, the peaks attributed to the substrate (Zr 3d) were still detected in the coated sample, even though with much lower intensities than those of pristine and cleaned samples. The change in intensity could be related to the topography of the nanocoating, with peak-to-valley distances larger than the average thickness measured by ellipsometry, rather than to the presence of pine-holes in the plasma polymer layer.

**Fig. 2:**
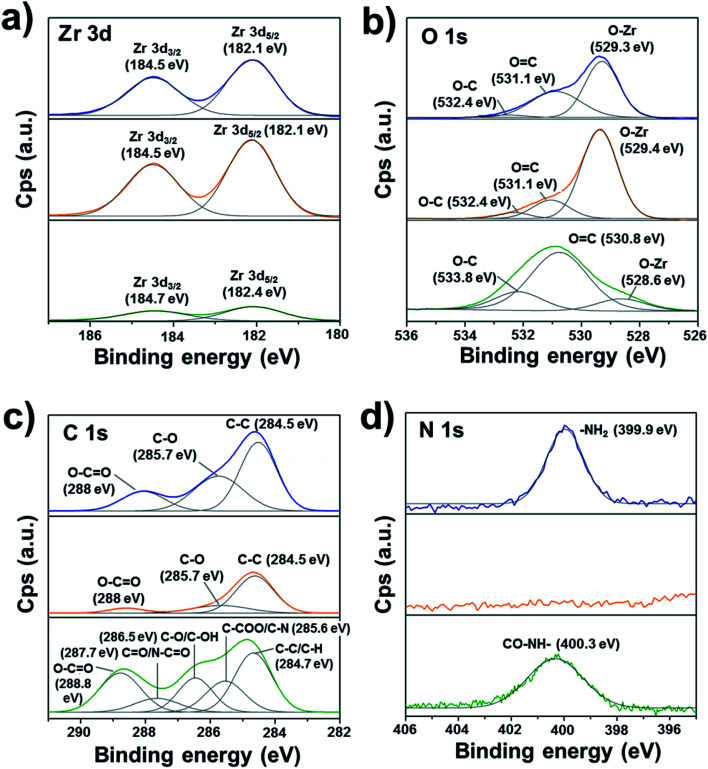
XPS high-resolution spectra of the zirconia surface before and after oxygen plasma treatment and coating with pPoly(EGDMA-*co*-DOMAm): (a) Zr 3d, (b) O 1s, (c) C 1s, and (d) N 1s.

XPS core level data was acquired for Zr 3d, O 1s, C 1s, and N 1s (Fig. 2). The deconvolution of Zr 3d_3/2_, Zr 3d_5/2,_ and O 1s XPS peaks did not show significant changes in the ZrO_2_ environment in the pristine zirconia and after oxygen plasma cleaning (Fig. 2a). On the contrary, a clear reduction of the contribution associated with the Zr–O bond in O 1s deconvoluted peaks (528.6–529.4 eV) was obtained (Fig. 2b). Moreover, the standard protocol for surface cleaning and activation of the zirconia disc successfully induced a reduction of the atmospheric organic contaminants present on the surface of the pristine discs (Fig. 2c and d). As expected, the organic character of the plasma polymer deposited layer was evidenced by a significant shift of C 1s and O 1s peaks intensities to higher binding energies and the occurrence of new components, such as C=O/N–C=O (287.7 eV), C–O/C–OH (286.5 eV) and C–COO/C–N (285.6 eV) in C 1s high-resolution spectrum of pPoly(EGDMA-*co*-DOMAm). Therefore, the contributions of the ester, amide, and catechol groups were successfully detected. The C/O ratio of 2.1 was slightly lower than the theoretical value of 2.5. In addition to the contribution of the oxygen from the ceramic, the formation of oxygen-containing polar groups is commonly attributed to polymer exposed to plasmas. Otherwise, the detection of nitrogen from DOMAm's amide groups (400.3 eV, Fig. 2d) was more efficient after cleaning the pristine zirconia surface with oxygen plasma.

The stability of the coating was studied by immersing zirconia discs in PBS buffer and further evaluation of the weight loss and the chemical structure composition by gravimetry and Raman analysis, respectively. Although the high hydrophilicity of pPoly(EGDMA-*co*-DOMAm) coatings (Section 3.2) would induce the polymer detachment by water absorption, the film adhered well to the zirconia surface and no delamination was observed, even after 7 days of immersion in the solution. Three replicates were analyzed by gravimetry and the film weight remained constant, confirming the efficiency of the plasma deposition.

**Fig. 3:**
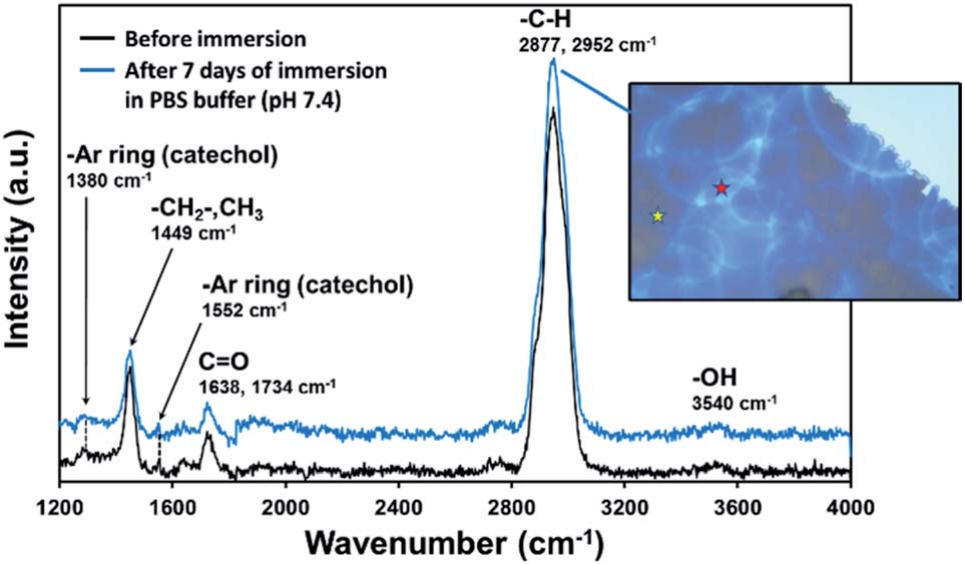
Raman spectra of zirconia disc decorated with pPoly(EGDMA-*co*-DOMAm) discs before and after immersion of samples in PBS buffer solution for 7 days. The inset represents the optical microscopy of the coating showing two zones analyzed after 1 week. The spectra of both points, indicated by yellow and red stars, are similar and only one of them has been plotted.

Raman spectra (Fig. 3) taken from two different zones after one week (Fig. 3, inset image) was comparable with that of samples freshly prepared. As can be seen, the main absorption bands appear in the range of 4000–1200 cm^−1^ (Raman region of 100–1200 cm^−1^ is not shown due to the substrate interference). The first broad and very strong absorption band at 2800–2950 cm^−1^ corresponds to C–H stretching (–CH_2_– and CH_3_ groups) and is confirmed by a fingerprint band at 1449 cm^−1^ (scissoring and asymmetric bending), which intensity is usually proportional to the number of methylene in the copolymer chain. Polar groups were also identified, showing low-intensity bands at 1552 cm^−1^ (–CH from aromatic rings) and at 1638 cm^−1^ and 1734 cm^−1^ (C=O). The two late peaks belong to amide II (–CONH–, DOMAm), which is much less intense than amide I, and to the ester group (–COO–, EGDMA), respectively. The absorption bands from catechol groups of PDA usually appear at ∼1350 cm^−1^ and ∼1580 cm^−1^ (stretching and deformation, respectively).^31^ Both absorption bands exhibit much lower intensity than the EGDMA groups (Fig. 3). Furthermore, the weak and broad band observed at 3540 cm^−1^, which corresponds to –OH stretching from catechol rings, confirms the presence of the DOMAm component. Our results are consistent with previous work, in which authors demonstrated the covalent linkage of hydroxyl groups from PDA with ZrO_2_ molecules.^37^ Thus, a lower intensity of catechol and hydroxyl groups from DOMAm in our system supposedly indicates a successful covalent bond of pPoly(EGDMA-*co*-DOMAm) with the substrate through aromatic and hydroxyl contributions.

### 3.2. Wettability and AFM investigations of zirconia/pPoly(EGDMA-*co*-DOMAm) surface

**Fig. 4:**
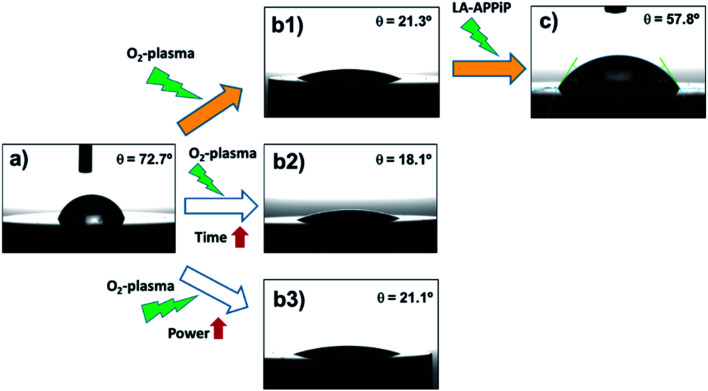
Variability of water contact angle, after oxygen plasma treatment and polymer deposition: (a) pristine zirconia disc; (b) zirconia disc after cleaning with oxygen plasma: (b1) 480 s + 1.6 W cm^−2^ plasma power (standard protocol, SP); (b2) 960 s + 1.6 W cm^−2^ plasma power (double time, DT); (b3) 480 s + 3.2 W cm^−2^ plasma power (double power, DP); and (c) zirconia/pPoly(EGDMA-*co*-DOMAm) disc. Filled arrows indicate the final route followed to prepare samples for biocompatibility assays.

The osseointegration of an implant is highly dependent on the following surface context: (i) wettability, (ii) roughness, and (iii) substrate chemical nature. First, the wettability of the samples was evaluated by comparing different surface treatments (Fig. 4). The pristine 3Y-TZP disc has hydrophilic interaction with water molecules, having a static WCA of 72.7° ± 2.0° (Fig. 4a). After oxygen plasma treatment (5%_v/v_ O_2_ in Ar) using a standard protocol (480 s at 1.6 W cm^−2^), the hydrophilicity increases by 70% (21.3° ± 1.5°, Fig. 4(b1)) with respect to the pristine sample, revealing the positive effect of the appearance of Zr–O species with charges (ZrO^−^, ZrOH^−^), and radical (Zr–O˙) groups induced by plasma. Kalyoncuoglu *et al.*^41^ also demonstrated the superhydrophilic behavior of polished zirconia substrates after O_2_-plasma, Ar-plasma, and CF_4_-plasma attack with a reduction of WCA of about 87%. On the contrary, an increase of O_2_-plasma time cleaning, by applying two sets of plasma discharges and maintaining the plasma power constant (DT protocol; 960 s at 1.6 W cm^−1^) (Fig. 4(b2)), or an increase of power force maintaining the time constant (DP protocol; 480 s at 3.2 W cm^−1^) (Fig. 4(b3)), do not affect the ceramic surface wettability. Thus, an almost unappreciated lowering of WCA values was observed (18.1° ± 1.9° and 21.1 ± 1.5°, respectively, for DT and DP plasma cleaning). Once the copolymer is generated by the LA-APPiP method, the WCA enhances (57.8° ± 2.2°, Fig. 4c), even though, as expected, it is still hydrophilic due to the polar chemical nature of the dopamine and acrylate monomers structure (Fig. 1).^23,24^

**Fig. 5:**
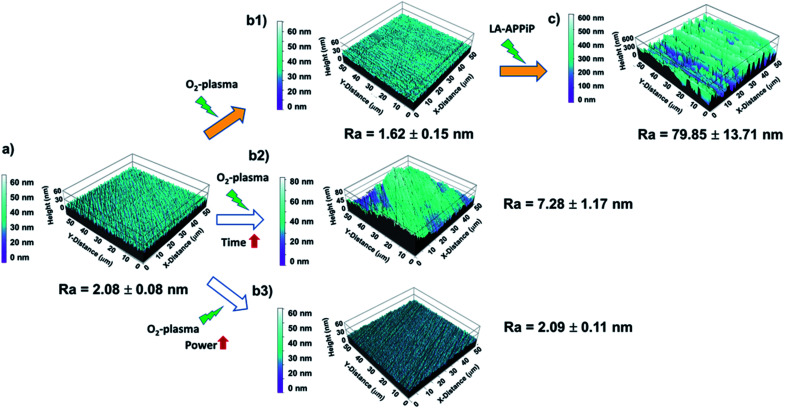
AFM topography images (50 μm × 50 μm) and roughness averages (*R*_a_) of zirconia discs with different pretreatments and after polymer deposition: (a) pristine zirconia disc; (b) zirconia disc after cleaning with oxygen plasma: (b1) 480 s + 1.6 W cm^−2^ plasma power (standard protocol, SP); (b2) 960 s + 1.6 W cm^−2^ plasma power (double time, DT); (b3) 480 s + 3.2 W cm^−2^ plasma power (double power, DP); and (c) zirconia/pPoly(EGDMA-*co*-DOMAm) disc. Filled arrows indicate the final route followed to prepare samples for biocompatibility assays.

Having checked that the new polymeric coating is stable in biological solution and is well-adhered, AFM studies were performed to evaluate the roughness and topography properties of Y-TZP/pPoly(EGDMA-*co*-DOMAm) biocomposite. Fig. 5 shows the evolution of the substrate interface after each cleaning step and after the polymerization process carried out by the LA-APPiP method.

Compared to laser-texturing ablation methods,^15,42,43^ which also help to enhance the wettability of the zirconia surface for cell adhesion, the O_2_-plasma surface treatment can be considered a “*gentle*” treatment. The zirconia surface can be considered “ultra-smooth” even after the application of double power discharge and increased plasma time, with a roughness average (*R*_a_) from 1.6 nm to 7.3 nm (Table 1) and comparable to the pristine surface (Fig. 5). Thus, the plasma cleaning does not affect the microstructure features of the zirconia surface. After the LA-APPiP of EGDMA and DOMAm, the *R*_a_ increased by a factor of 3.9 with respect to the plasma cleaned or polished zirconia (79.9 ± 13.7 nm). The nanometric polymer layer is homogeneously distributed in an area of 50 × 50 μm^2^ of the substrate, as can be seen in Fig. 5c. Moreover, SEM images did not show any defect for larger areas in the topography analyses (Fig. S2†).

**Table 1:** Root-mean-square roughness (*R*_q_) and roughness average (*R*_a_) values as obtained by AFM after different oxygen-plasma pre-treatment conditions and after plasma polymerization

Plasma treatment	*R*_q_ (nm)	*R*_a_ (nm)
Pristine sample (polished)	2.63 ± 0.14	2.08 ± 0.08
Standard protocol (SP)	2.21 ± 0.25	1.62 ± 0.15
Double time protocol (DT)	9.13 ± 1.32	7.28 ± 1.17
Double power protocol (DP)	2.72 ± 0.17	2.09 ± 0.11
pPoly(EGDMA-*co*-DOMAm) (plasma polymerized coating)	94.61 ± 13.93	79.85 ± 13.71

The formation of a wrinkle-like topography might be attributed to the dynamic deposition (layer-after-layer) of well-adherent crosslinked polymeric layers from very thin liquid layers.^44^ The difference between the first layers and the consecutive ones generates surface stress due to a mismatch of their elastic properties, being the initial layers less elastic due to their adhesion to the much more rigid zirconia surface. The results altogether support the previous statement of a well-adherent biocompatible pPoly(EGDMA-*co*-DOMAm) nanometric film.

Therefore, the best route used for the final zirconia/pPoly(EGDMA-*co*-DOMAm) discs preparation was that expressed as (a), (b1), and (c) in Fig. 4. The O_2_-plasma cleaning has been demonstrated to be a crucial preliminary step for the obtaining of well-adhered covalently bonded pPoly(EGDMA-*co*-DOMAm) copolymer film on zirconia surfaces.

### 3.3. *In vitro* biocompatibility

In this study, two different materials, a zirconia substrate and a nanocoating of a biocompatible copolymer, with different surface properties (as shown before), have been investigated for their biocompatibility with MG-63 human cells. The greater value of roughness leads to better wettability of the surface and better bonding properties of the material with other systems.

The response of PDA self-polymerized on plastics, titanium, and ceramic substrates (including zirconia) with enhanced osteoblastic adhesion towards cells has been extensively investigated using *in vitro* assays.^29,30,36,45^ However, deep research on the effect of PDA and derivatives synthesized by atmospheric plasma was never approached.

**Fig. 6:**
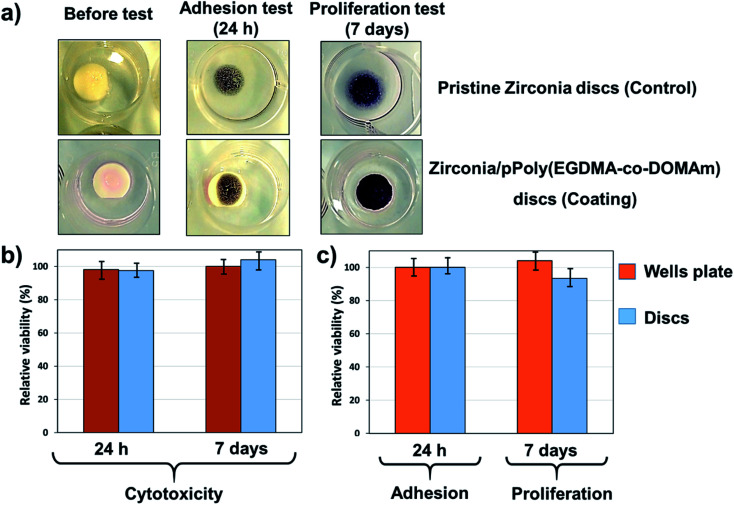
Viability of osteogenic MG-63 cells with zirconia/pPoly(EGDMA-*co*-DOMAm) decorated discs: (a) optical microscopy images of TCP wells plate with pristine and zirconia/pPoly(EGDMA-*co*-DOMAm) discs, before and after MG-63 cells incubation; (b) cytotoxicity evaluation after 24 h and 7 days; and (c) adhesion of MG-63 cells after 24 h and cells proliferation after 7 days, respectively.

As expected, the adhesion and proliferation were different. Fig. 6 demonstrates that the pPoly(EGDMA-*co*-DOMAm) copolymer surfaces support adhesion and proliferation of osteogenic MG-63 cells, having adherent cells of fibroblast type. The macroscopic image of the pPoly(EGDMA-*co*-DOMAm) copolymer discs seeded with MG-63 cells onto the material surface (Fig. 6a) shows the reaction of viable cells with the MTT reagent. The reaction corresponds to the conversion of the MTT reagent into a dark blue formazan salt. Dehydrogenase enzymes mediate such reactions from mitochondria and lysosomes in viable cells. In this way, the stain of the discs is similar to the stain observed on the culture plates, which correspond to the control. These salts can then be dissolved in some organic solvents, *e.g.*, DMSO, and allow quantification of viable cells (as described in the experimental section).

Also, the viability of the cells on the surface of the material and in the total well of the culture plate was quantitatively determined. The latter measurement indicates the possible cytotoxicity effects induced by the release of the material or its products (*i.e.* monomer, dissolvent, *etc.*) to the culture medium. Fig. 6b shows that the cell adhesion evaluated after 24 h of culture on the surface of the pPoly(EGDMA-*co*-DOMAm) copolymer was similar to the control, and it is also shown that the material does not have any cytotoxic effect when adhesion is evaluated in the total well (cells attached to the material plus cells attached to the plate). Cell proliferation (Fig. 6c), which is evaluated after 7 days of culture, reflects the capacity of cell division to increase the number of cells and material colonization. Results show that the pPoly(EGDMA-*co*-DOMAm) copolymer allows cell growth and colonization on its surface. Moreover, it was also demonstrated that in prolonged time (7 days), the samples lack any cytotoxic effect.

**Fig. 7:**
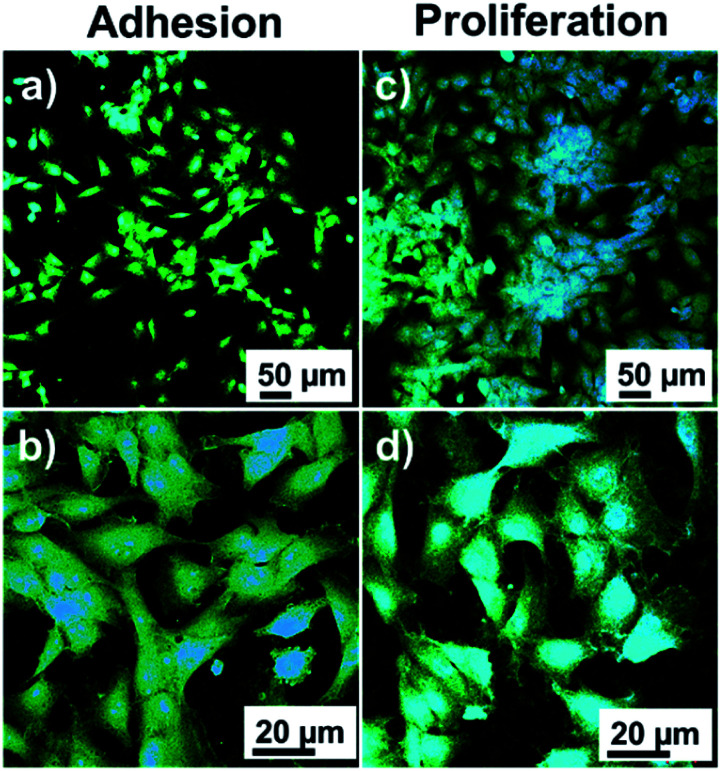
Fluorescence optical images of osteogenic MG-63 cells adhered to zirconia/pPoly(EGDMA-*co*-DOMAm) discs: (a and b) low and high magnification images of the adhered cells, after 24 h of incubation, respectively; and (c and d) low and high magnification images of cells proliferation, after 7 days of incubation, respectively. MG-63 cells were stained with phalloidin dye, in which the nucleus is represented as blue color, and the cytoplasmic actin filaments are marked as green color.

Fluorescence microscopy images of MG-63 cells are shown in Fig. 7. The cells in extension on the surface of the material are clearly observed during adhesion and cells in higher density during growth. High magnification micrographs show that in both cases the cells extend their cytoplasm-shaped filopodia to adhere to the surface and promote cell migration in the cell adhesion and establish contacts between cells. These results prove that the catechol/acrylate copolymer has biocompatible characteristics *in vitro* and allows the adhesion and growth of osteogenic MG-63 cells.

## 4. Conclusions

For the first time, a poly(EGDMA-*co*-DOMAm) copolymer coating has been successfully covalently bonded to ceramic substrate. The employment of atmospheric plasma to activate the zirconia surface and the liquid-assisted polymerization of polydopamine and ethylene glycol dimethacrylate monomers by plasma (called LA-APPiP) resulted in a fast and friendly way to obtain a very smooth and stable nanometric film. The new ceramic coating has enhanced human cell proliferation and adhesion, after 24 h and 7 days of incubation, respectively; when compared to the pristine zirconia samples.

Such results open new insights for continuing the investigations of applying LA-APPiP method to replace less friendly approaches, like layer-by-layer deposition, sol–gel technologies, or adsorption methods, for the obtaining of thin films in solid substrates; which usually expend a lot of solvents and purification steps. LA-APPiP can be extended to other materials surfaces, such as polymers, metals, and other ceramic compounds, and can be easily scalable for industrial applications.

## Conflicts of interest

There are no conflicts to declare.

## Supplementary Material

RA-011-D1RA02054D-s001
