# Supplementary material for: Atmospheric pressure plasma liquid assisted deposition of polydopamine/acrylate copolymer on zirconia (Y-TZP) ceramics: a biocompatible and adherent nanofilm
Source: RSC Adv. 2021 May 11;11(28):17360–8. doi: 10.1039/d1ra02054d (PMC9033174; doi:10.1039/d1ra02054d)
Supplement: RA-011-D1RA02054D-s001 [file RA-011-D1RA02054D-s001.pdf]

Electronic Supplementary Material (ESI) for RSC Advances.
This journal is © The Royal Society of Chemistry 2021

Supporting file

Atmospheric pressure plasma liquid assisted deposition of polydopamine/acrylate copolymer on zirconia (Y-TZP) ceramics: a biocompatible and adherent nanofilm

Ľudmila Hodásová,[1,2,3] Robert Quintana,[4] Urszula Czuba,[4] Luis J. del Valle,[1,3] Gemma Fargas,[2,3] Carlos Alemán,[1,3] Elaine Armelin[1,3*]*

[1] Departament d'Enginyeria Química, Universitat Politècnica de Catalunya, Campus Diagonal Besòs (EEBE), C/ Eduard Maristany, 10-14, Building I, 2nd floor, 08019, Barcelona, Spain.

[2] Departament de Ciència i Enginyeria de Materials, Universitat Politècnica de Catalunya, Campus Diagonal Besòs - EEBE, C/ Eduard Maristany, 10-14, Building I, 1st floor, 08019, Barcelona, Spain.

[3] Barcelona Research Center for Multiscale Science and Engineering, Universitat Politècnica de Catalunya, Campus Diagonal Besòs (EEBE), C/ Eduard Maristany, 10-14, Building I, basement floor, 08019, Barcelona, Spain.

[4] Luxembourg Institute of Science and Technology (LIST), Materials Research and Technology Department, L-4422 Belvaux, Luxembourg.

Corresponding authors: elaine.armelin@upc.edu and roberto.quintana@list.lu

3. Results and discussion

3.1. Success of atmospheric-pressure plasma deposition of poly(EGDMA-*co*-DOMAm) in zirconia substrates activated by oxygen plasma

Table S1. Chemical composition and main properties of 3Y-TZP discs.

Chemical composition	ZrO_2	Y_2O_3	Al_2O_3
	94.55 %	5.2 %	0.25 %
Sintering temperature	1450 ºC		
Density	6.05 g/cm^3		
Hardness	14-15 GPa		
Young's modulus	230 GPa		
Dielectric constant ［1 MHz］	32.7		
Dielectric loss ［1 MHz］	0.006		
Thermal conductivity	3 W/(m·K)		
Thermal expansion	9.6 × 10^{-6} m/ºC (25–300 °C)		

Table S2. XPS atomic composition of the zirconia disc sample coated with pPoly(EGDMA-*co*-DOMAm) by atmospheric plasma deposition.

Element	Atomic concentration (%)
**C 1s**	63.0
**O 1s**	30.7
**N 1s**	1.4
**Zr 3d**	4.3
**Y 3d**	0.6

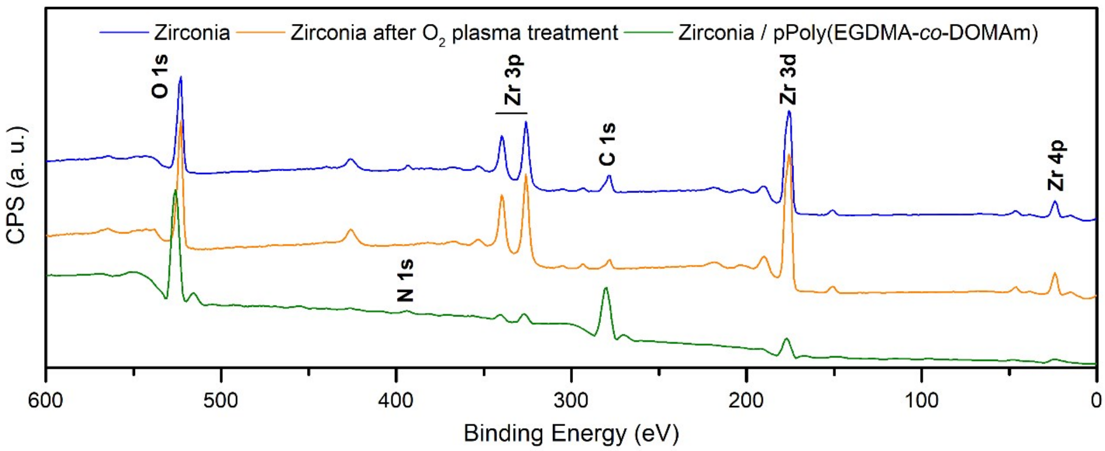

Figure S1. Survey XPS spectra of the pristine zirconia discs (upper plot), after standard oxygen plasma treatment protocol (SP) (middle plot) and coated with pPoly(EGDMA-*co*-DOMAm) (bottom plot).

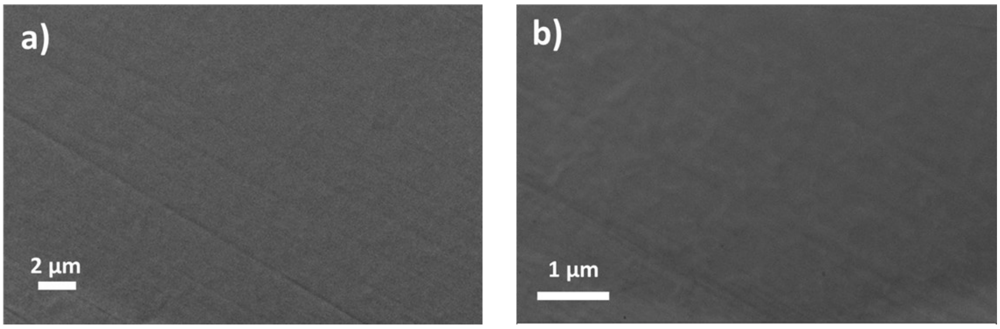

Figure S2. SEM micrographs taken from the pPoly(EGDMA-*co*-DOMAm) film surface, after LA-APPiP polymerization: a) 5 kX of magnification and b) 15 kX of magnification.
